# Postprandial Glycemic and Insulinemic Effects of the Addition of Aqueous Extracts of Dried Corn Silk, Cumin Seed Powder or Tamarind Pulp, in Two Forms, Consumed with High Glycemic Index Rice

**DOI:** 10.3390/foods8100437

**Published:** 2019-09-24

**Authors:** Sumanto Haldar, Linda Gan, Shia Lyn Tay, Shalini Ponnalagu, Christiani Jeyakumar Henry

**Affiliations:** 1 Clinical Nutrition Research Centre (CNRC), Singapore Institute for Clinical Sciences (SICS), Agency for Science Technology and Research (A*STAR), 30 Medical Drive, Singapore 117609, Singapore; lindaalerts@gmail.com (L.G.); tay_shia_lyn@sics.a-star.edu.sg (S.L.T.); shalini_Ponnalagu@sics.a-star.edu.sg (S.P.); jeya_henry@sics.a-star.edu.sg (C.J.H.); 2 Department of Biochemistry, National University of Singapore, Singapore 117596, Singapore

**Keywords:** corn silk, cumin, tamarind, aqueous extracts, form, postprandial glycemia, postprandial insulinemia

## Abstract

Several plant-based traditional ingredients in Asia are anecdotally used for preventing and/or treating type 2 diabetes. We investigated three such widely consumed ingredients, namely corn silk (CS), cumin (CU), and tamarind (TA). The aim of the study was to determine the effects of aqueous extracts of these ingredients consumed either as a drink (D) with high-glycemic-index rice or added to the same amount of rice during cooking (R) on postprandial glycemia (PPG), insulinemia (PPI), and blood pressure (BP), over a 3 h measurement period. Eighteen healthy Chinese men (aged 37.5 ± 12.5 years, BMI 21.8 ± 1.67 kg/m^2^) took part in a randomized crossover trial, each completing up to nine sessions. Compared to the control meal (plain rice + plain water), the addition of test extracts in either form did not modulate PPG, PPI, or BP. However, the extracts when added within rice while cooking gave rise to significantly lower PPI than when consumed as a drink (*p* < 0.01). Therefore, the form of consumption of phytochemical-rich ingredients can differentially modulate glucose homeostasis. This study also highlights the need for undertaking randomized controlled clinical trials with traditional foods/components before claims are made on their specific health effects.

## 1. Introduction

Arising from traditional dietary and alternative medicine practices across Asia (e.g., traditional Chinese medicine, Ayurveda) there have been a plethora of food-based components claimed as beneficial in improving the risk of diabetes and other cardiometabolic conditions. Unfortunately, the majority of these traditional practices have been based on historical pretext rather than on solid scientific evidence. In fact, robustly controlled clinical trials have been sparse, and most of the research claims came from in vitro studies and/or in vivo animal studies. In general, these traditional ingredients are all plant-based ingredients, rich in polyphenols and other phytonutrients with a wide range of biological functions including α-amylase and α-glucosidase enzyme inhibition, anti-inflammatory and antioxidant properties, insulin secretagogues, etc. [1,2,3]. In Asia, over centuries these ingredients have been integrated as part of daily diets of individuals into various culinary preparations and thereby used simultaneously as traditional “medicines” to maintain health and reduce disease risk. Three such commonly used traditional ingredients include corn silk, cumin, and tamarind, and some of the prior evidence for their biological effects related to improving glycemic control and/or type 2 diabetes risk are outlined below.

Corn silk (*Stigma maydis*) is a waste by-product of corn and has been long used in traditional Chinese medicine to treat diabetes and associated conditions [4]. Corn silk is rich in various phytonutrients (e.g., polyphenols, terpenoids, etc.), vitamins, and minerals, as detailed elsewhere [5]. There are several mechanisms through which corn silk is thought to improve glycemic control, which include inhibition of α-amylase and α-glucosidase enzymes [6,7,8], inhibition of inflammation [9], reduced formation of advanced glycation end products (AGEs), reduced oxidative stress, and the enhancement of glucose-stimulated insulin secretion [6,10,11]. Therefore, corn silk has the potential to improve postprandial glycemic control, although to our best knowledge this has not been tested using a randomized controlled trial (RCT) in normoglycemic, non-diabetic individuals.

Similarly, both cumin and tamarind have been used as traditional food ingredients across various Asian cuisines and are considered to have glycemic-improving properties. For example, it has been previously shown that the consumption of cumin seeds by streptozotocin-induced diabetic rats led to significant reductions in hyperglycemia and glucosuria as well as improvements in renal function [12]. Another study showed reductions in both fasted blood glucose and glycated hemoglobin when alloxan-induced diabetic rats were administered cumin over a 6-week period [13], whereas a separate study showed a reduction in postprandial glycemic responses in rabbits fed cumin [14]. More recently, it has also been demonstrated that the cuminaldehyde found in cumin has significant α-glucosidase as well as aldose reductase inhibition [15]. On the other hand, tamarind pulp extract has been shown to improve glycemic, insulinemic, as well as lipidemic responses upon feeding to high-fat-induced obese rats for 10 weeks [16]. Several other studies using rat/mice models of diabetes have also shown improvements in glycemic parameters with various extracts of tamarind [17,18,19,20]. While the collective evidence in animal studies for both cumin and tamarind having the potential to improve glycemic control is rather substantial, there have been hardly any randomized controlled trials in humans.

Therefore, in this “Phytochemical Rich Ingredients as Functional Foods (PRIFF)” study, the primary aims were to assess the three ingredients, viz., corn silk, cumin, and tamarind as listed above, in terms of their effects on postprandial glycemia and insulinemia, when consumed with a carbohydrate-rich meal principally consisting of a high-glycemic-index (GI) white rice. The secondary aim was to also measure the effects of these three ingredients on postprandial blood pressure. The study used aqueous extracts which can be obtained using household preparation methods. For cumin and tamarind, we used dietary doses, whereas for corn silk we used two separate doses—the “high dose” consisting of an amount commonly used in traditional Chinese medicine, and a lower (one-half of the high) dose to assess whether this lower, “dietary” dose would still be effective in improving glycemic parameters. Moreover, there is now considerable evidence that the food form plays an important role in determining glycemic and/or insulinemic responses [21,22,23]. We therefore investigated all the test ingredients in two separate forms: (i) extracts consumed as a drink (D) with plain rice or (ii) extracts added into rice (R) during cooking and consumed with plain water. 

## 2. Materials and Methods

### 2.1. Recruitment and Ethics

Eighteen volunteers were enrolled in this study through flyers placed around The National University of Singapore and from our center’s recruitment database of volunteers who had previously given consent to be contacted for research. Inclusion criteria for this study were: Chinese, male, age between 21 to 60 years old, body mass index (BMI) between 18.5 and 25.0 kg/m^2^, waist circumference ≤90 cm, fasting blood glucose <7.0 mmol/L, blood pressure <140 mmHg and <90 mmHg for systolic and diastolic, respectively. Interested volunteers attended a screening visit after an overnight fast (no food or drink except water for a minimum of 10 h). At the screening visit, height was measured using a stadiometer (Seca 217, Hamburg, Germany), weight and body fat percentage were measured using the Tanita analyzer (BC-418, Tokyo, Japan), seated resting blood pressure was measured using an automated sphygmomanometer (Omron HEM907, Singapore). Fasting glucose was measured in whole blood using a HemoCue 201 RT analyzer (Angelholm, Sweden) upon obtaining capillary blood using a single-use lancet device (Abbott Diabetes Care, Alameda, CA, USA). Volunteers were excluded if they met any one of the following criteria: taken part in sports at the competitive and/or endurance levels; smoking; being allergic to common food (e.g., eggs, shellfish, dairy, nuts, gluten) and test ingredients; intentionally restricting food intake; have glucose-6-phosphate deficiency (G6PD) or metabolic diseases, cardiovascular diseases, or disorders involving the gut, liver, or kidney; taking prescribed medication or dietary supplement that affects glycemia or interferes with other study measurements; consume ≥6 alcoholic drinks per week; donated blood within 4 weeks of study participation; had poor veins impeding venous access; had a history of severe vasovagal syncope following blood draws; had or was a carrier of a chronic infection (e.g., hepatitis B or C, human immunodeficiency virus); suffering from active tuberculosis (TB) or receiving treatment for TB; or was related to a member of the study team. All volunteers provided written informed consent. All volunteers were given a choice of attending five or nine test sessions. This study was reviewed and approved by the National Healthcare Group (NHG) Domain-Specific Review Board, Singapore (DRSB Ref: 2018/00258). The study has been registered on clinicaltrials.gov under study ID No. NCT03685916. 

### 2.2. Preparation of Aqueous Extracts and Study Meals

Dried, mature corn silk, commonly sold as an ingredient to treat diabetes by traditional Chinese medicine (TCM) practitioners, was purchased locally. Aqueous extracts were obtained by adding 552 g of corn silk to 2.3 L of tap water and boiling for 15 min. A total of three batches of extraction were undertaken. After pooling, the total volume of the corn silk extract (CS) was made up to 6.9 L and filtered through a fine mesh strainer to remove corn silk particles. Then, 50 mL of this concentrated extract (24 g/100 mL) was aliquoted and stored frozen in a −20 °C freezer until further use. An aqueous extract of cumin was prepared by adding 552 g of ground cumin powder (Pattu brand, Sabi Foods (India) Pvt. Ltd., Tamin Nadu, India) into 2.4 L of tap water and allowing the mixture to boil for 15 min. A further 2.4 L of tap water was added to the viscous mixture and boiled for an additional 15 min. Finally, the cumin extract (CU) was filtered through a fine mesh filter and the volume made up to 2.3 L and filtered through a fine mesh strainer to remove cumin particles. Then, 50 mL of this concentrated extract (24 g/100 mL) was aliquoted and stored frozen in a −20 °C freezer until further use. For the tamarind extract (TAM), 460 g of deseeded tamarind pulp (Lion Dates Impex Pte Ltd., Trichy, India) was added to 1.2 L of tap water and boiled for 15 min. The first aqueous extract was obtained by filtering the mixture through a fine mesh filter and the residual tamarind pulp re-extracted with another 1.2 L of tap water via boiling for another 15 min. The mixture from the second extraction was filtered and both tamarind aqueous extracts were pooled and the volume made up to 2.3 L. Then, 50 mL of this concentrated extract (20 g/100 mL) was aliquoted and stored frozen in a −20 °C freezer until further use. All stored concentrated extracts were thawed and diluted either four-fold for CS-12, CU, and TAM, or diluted two-fold for CS-24 with filtered water on the day of the test session, to make up to a total of 200 mL of “diluted aqueous extract”.

The study meals (including rice) were prepared fresh every morning, and the test extracts were either added into rice (R) during cooking or made up into a drink (D). The study meals were rich in carbohydrates, providing approximately 50 g of available carbohydrates mainly from high-GI glutinous rice (Thai glutinous rice, Fairprice, Singapore) and 20 g of cooked garden peas (Frozen garden peaks, Fairprice, Singapore). The control meal (CON) was made up of 65 g of glutinous rice cooked in 200 mL of filtered water in a household rice cooker, 20 g of peas (steamed for 2 min in microwave), and a glass (200 mL) of filtered water. The rice (R) test meals consisted of 60 g of glutinous rice cooked in 200 mL of the diluted aqueous extract (detailed above), 20 g of peas, and 200 mL of filtered water, whereas the drink (D) test meals consisted of 60 g of glutinous rice cooked in 200 mL of plain filtered water, 20 g of peas, and 200 mL of diluted aqueous extract. The food ingredients used and the energy content and nutrient composition of each test meal as obtained from food packet labels or nutrient databases (US Department of Agriculture) are shown in Table 1. 

### 2.3. Total Polyphenol Content (TPC Analyses) of Aqueous Extracts

Total polyphenol contents (TPCs) were determined by the Folin–Ciocalteu (FC) assay in triplicates using a method adopted from Medina-Remón et al. [24]. Briefly, 1 mL of the aqueous concentrated extract was transferred to a microfuge tube and centrifuged at 13,000× *g* for 5 min at room temperature to obtain the supernatant. Subsequently, 100 µL of tap water (blank), supernatant, or gallic acid standard was added to 200 µL of 10% FC reagent in a 96-well clear polystyrene microplate followed by the addition of 800 µL of 700 mM sodium carbonate. After a 2-h incubation at room temperature in the dark, the absorbance at 765 nm was measured in a microplate reader (Tecan Infinite 200, Mannedorf, Switzerland). The TPC was calculated using the regression equation derived from a gallic acid standard curve and expressed as gallic acid equivalents (GAE).

### 2.4. Study Design

**Figure 1:**
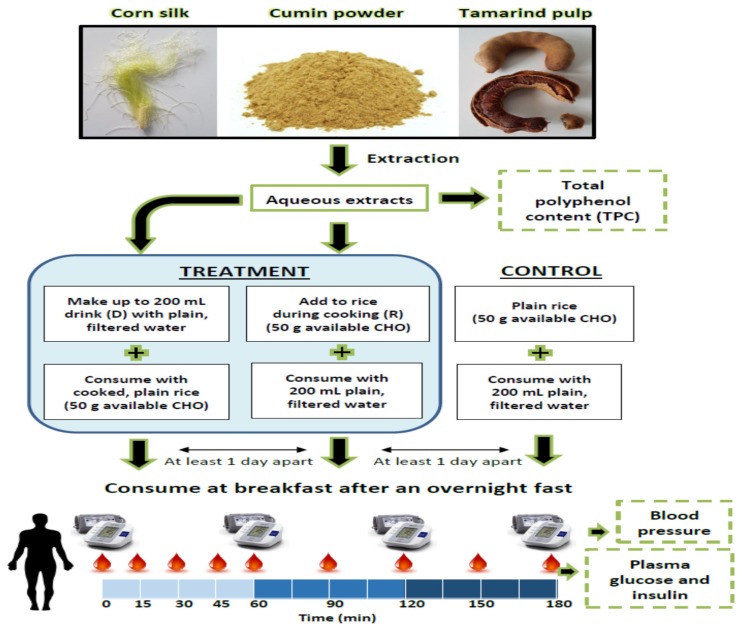
Schematic of study design.

This was a randomized crossover design whereby each volunteer came for five or nine test sessions with a minimum 1-day wash-out between sessions. The order of the study meals were randomized using the RAND function in Microsoft Excel. Volunteers were asked to avoid strenuous exercise and alcohol intake 24 h prior to their test session and to arrive at the center at 08:30 after an overnight fast of at least 10 h. Upon arrival, volunteers had their baseline (T0) blood pressure measured and a fasted blood sample drawn (after 5 min of rest) at the antecubital vein via cannula by a trained phlebotomist. Volunteers were then served the study meal and instructed to consume the meal within 15 min. Subsequent blood collection was carried out at T15, 30, 45, 60, 90, 120, 150, and 180 min after the first bite. Duplicate blood pressure measurements were taken hourly at T60, 120, and 180 min. A schematic of the study design is shown in Figure 1. 

### 2.5. Blood Sampling and Analytical Methods

Venous blood was collected in K_2_EDTA vacutainers (BD, New Jersey, NJ, USA), placed immediately on ice, and centrifuged at 1500× *g* for 15 min within an hour of blood draw to obtain plasma. Plasma samples were then stored at −80 °C in aliquots until ready for analyses. Plasma glucose and insulin concentrations were determined by the COBAS c311 and e411 automated analyzers (Roche Diagnostics GmbH, Berlin, Germany), respectively. For each analyzer, a successful two-point calibration was performed at the start of the study. Thereafter, quality control was done daily before analyzing the plasma samples. For each assay, 250 µL of thawed undiluted plasma was analyzed in a Hitachi cup using the manufacturer’s recommended cassette for glucose and insulin measurement. All samples were analyzed over a 5-day period using the same analytical instrumentations and laboratory consumables (including standards and quality controls). All samples from a given individual (i.e., all treatments and all time points) were analyzed within the same batch and the same day, in order to limit any effects of experimental variations. The inter-batch coefficient of variation for glucose analysis was 7.51%, and that for insulin analysis was 1.99%. 

### 2.6. Statistical Analyses

The primary outcome of this study was the acute effects of the test ingredients on the postprandial glucose and insulin responses for up to 180 min. The secondary outcomes were: i) the effect of the form in which the test ingredients were administered (R vs. D) on the 30, 120, and 180 min postprandial glucose and insulin responses; and ii) the acute effects of the test ingredients on the postprandial blood pressure measured over 180 min. Incremental areas under the curve (iAUCs) of glucose and insulin were calculated using the change in glucose or insulin above baseline fasting concentration, ignoring the area beneath the baseline. Total areas under the curve (tAUCs) of systolic and diastolic blood pressure measurements were calculated using the absolute blood pressure values measured at the time points.

All analyses in this study were done using Statistical Package for the Social Sciences (SPSS) version 24 (IBM, New York, NY, USA). Data were assessed for normality using the Shapiro–Wilk test as well as visually from the histogram and normal Q–Q (quantile-quantile) plots. Square-root transformation of the data was used where necessary to achieve normality. The treatment effect on the iAUCs of insulin and glucose was tested using linear mixed effects procedure in SPSS with treatment as the fixed factor and compound symmetry (CS) covariance structure. For the interaction between form and treatment test, a linear mixed effects procedure with both treatment and form as fixed factors was used with CS covariance structure. 

## 3. Results

Mean total polyphenol content (TPC) was lowest in the cumin aqueous extract (103.0 ± 3.4 mg GAE/100 g of cumin powder) and highest in the tamarind aqueous extract (440.1 ± 13.3 mg GAE/100 g of tamarind pulp). Mean TPC of corn silk was 233.1 ± 2.2 mg GAE/100 g of dried corn silk.

Of the 18 volunteers enrolled in the study, 16 volunteers completed all nine test sessions. One volunteer only completed three test sessions, while another only completed five sessions. The baseline anthropometric and metabolic characteristics of the volunteers to indicate the profile of volunteers who completed the study are presented in Table 2. All participants had their fasted glucose concentration at screening visit of less than 6.0 mmol/L, except for one participant who had a concentration of 6.20 mmol/L and would therefore be considered as having impaired fasted glucose (IFG). 

### 3.1. Postprandial Glycemic and Insulinemic Response to Control and Test Meals

**Figure 2:**
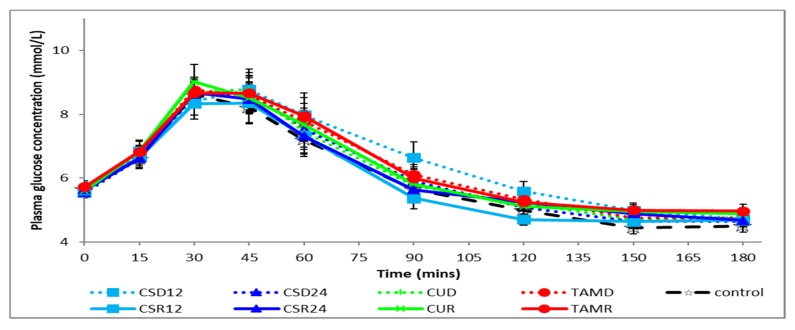
Postprandial changes in plasma glucose concentration during various treatments. CSD12: corn silk drink 12 g; CSR12: corn silk rice 12 g; CSD24: corn silk drink 24 g; CSR24: corn silk rice 24 g; CUD: cumin drink; CUR: cumin rice; TAMD: tamarind drink; TAMR: tamarind rice.

**Figure 3:**
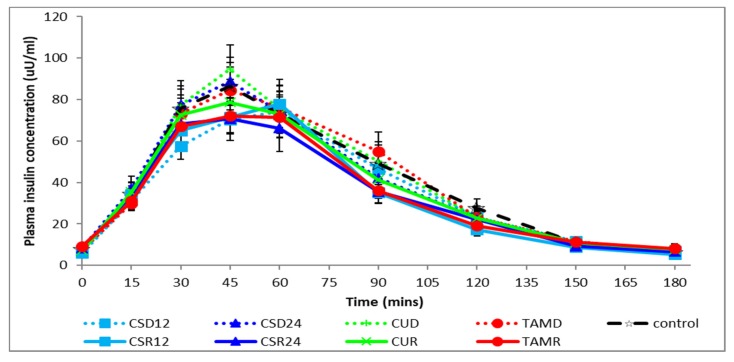
Postprandial changes in plasma insulin concentration during various treatments. CSD12: corn silk drink 12 g; CSR12: corn silk rice 12 g; CSD24: corn silk drink 24 g; CSR24: corn silk rice 24 g; CUD: cumin drink; CUR: cumin rice; TAMD: tamarind drink; TAMR: tamarind rice.

Figure 2 and Figure 3 respectively show plasma glucose and insulin concentrations over time. As expected, mean plasma glucose concentration rose significantly above baseline fasting levels in the first hour after meal initiation, peaking at T30 and falling below baseline at T150 and T180 (Figure 2). Similarly, the mean plasma insulin concentration rose significantly above baseline fasting levels in the first 2 h after meal initiation, peaking at T45 and returning to baseline levels at T150 (Figure 3). There were no significant differences between the various treatments as compared with the control on postprandial glycemia, irrespective of the food form, evaluated using incremental areas under the curve for various time intervals (iAUC_30_, iAUC_120_, or iAUC_180_), as shown in Table 3. There were also no differences in the postprandial glycemic responses between the two different forms (rice vs. drink) of consumption, irrespective of the test ingredients. Similarly, there were no significant differences in the influence of the various treatments as compared with the control on postprandial insulinemia as evaluated using incremental areas under the curve for various time intervals (iAUC_30_, iAUC_120_, or iAUC_180_). However, there was a significant increase (*p* < 0.01) in postprandial insulinemia (iAUC_120_ or iAUC_180_) when the test ingredients were consumed as a drink (D) as compared with the same ingredients being cooked within rice (R), irrespective of the test ingredients. It should also be noted that the form × treatment interactions were not significant across all measures, as shown in Table 3.

### 3.2. Postprandial Changes in Blood Pressure during Control and Test Meals

**Figure 4:**
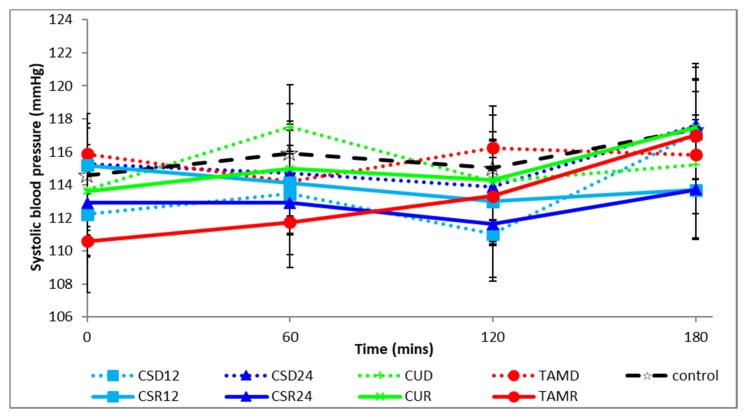
Postprandial changes in systolic blood pressure during various treatments. corn silk drink 12 g; CSR12: corn silk rice 12 g; CSD24: corn silk drink 24 g; CSR24: corn silk rice 24 g; CUD: cumin drink; CUR: cumin rice; TAMD: tamarind drink; TAMR: tamarind rice.

**Figure 5:**
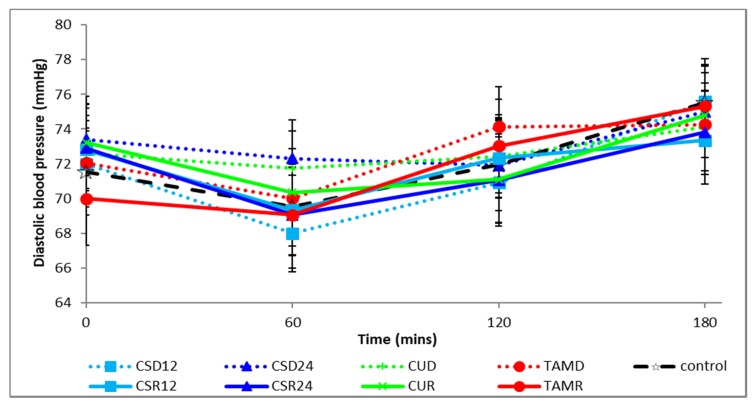
Postprandial changes in diastolic blood pressure during various treatments. CSD12: corn silk drink 12 g; CSR12: corn silk rice 12 g; CSD24: corn silk drink 24 g; CSR24: corn silk rice 24 g; CUD: cumin drink; CUR: cumin rice; TAMD: tamarind drink; TAMR: tamarind rice.

The postprandial changes in systolic and diastolic blood pressures are shown in Figure 4 and Figure 5, respectively. There were no significant differences between treatments, irrespective of form or between forms, irrespective of treatments at baseline and in the total area under the curve (tAUC) of the postprandial systolic or diastolic blood pressure, as shown in Table 4.

## 4. Discussion

Given that Asians are at a greater risk of type 2 diabetes and prediabetes, and considering that the dietary carbohydrate loads in Asians are much higher than in other parts of the world, there is a drive to identify functional ingredients within the Asian dietary context which can improve glycemic control. This trial was set out to test the effects of three separate ingredients, at dietary doses, for their individual effects on postprandial glycemia and insulinemia when consumed along with a carbohydrate-rich meal consisting of a portion of high-GI rice. We have previously shown improvements in postprandial glycemic control with polyphenol-rich mixed spices [25,26]. The three ingredients chosen in this study were also rich in phenolic compounds as measured here and reported elsewhere for cumin [27,28,29], tamarind [30,31,32,33], and dried corn silk [5,34]. Furthermore, inhibition of α-amylase and α-glucosidase activities are important determinants for any natural compound with a potential to reduce postprandial glycemia [35], and all three ingredients used in our study (or compounds found within them) have been shown to possess such activities in previous studies [8,36,37,38,39]. These ingredients were also specifically chosen for their extensive traditional utility, particularly across Asia. Cumin and tamarind are both used in several preparations as spices/condiments in rice-based dishes (e.g., “*jeera*” (cumin) rice), curries, soups (e.g., cumin and/or tamarind in “*rasam*”, mulligatawny soup, tom yum goong soups, etc.) or as beverages such as “*jal jeera*” (cumin water in India) or as “*nam makham*” (tamarind drink in Thailand). Given their extensive consumption in several different forms (i.e., in foods and in beverages), we further investigated any potential differences in their glycemic/insulinemic responses when either consumed as a drink or added into rice while cooking. Corn silk extract (CS) on the other hand was investigated not only due to its extensive use by traditional Chinese medicine practitioners as an anti-diabetic formulation [4,40], but also because corn silk is a common agricultural waste product which is rich in polyphenols and there is potential utility of its reintroduction back into the food system. This is particularly relevant considering the recent drive to re-introduce polyphenol-rich food-processing by-products back into the food system, as had been discussed in detail elsewhere [41].

Despite our careful selection of the ingredients tested, our study failed to demonstrate any significant effects on postprandial glycemia or insulinemia as compared to the control meal without these ingredients. There could be several reasons for this observation. First of all, we recruited non-diabetic, healthy individuals, all of whom are likely to have had adequate glycemic control. Indeed, some studies have previously shown that the postprandial glycemic response even between high- and low-GI meals did not differ in young healthy individuals [42]. Furthermore, the postprandial metabolic responses to the same foods are often different between normal and at-risk individuals [43,44]. Therefore, the results of our study may not be applicable to type 2 diabetic or other at-risk populations, who may well have benefited from the ingredients used. Nonetheless, the strengths of our study were that we used a crossover design, carefully matched the macronutrient composition between treatments, and used dietary doses of the ingredients in a real-life dietary context. In contrast, most of the animal studies supporting favorable effects often used much higher relative doses which are not achievable through usual dietary means in humans. Nonetheless, considering that our ingredients were rich in various phytochemicals with antioxidant as well as anti-inflammatory properties, there may be other favorable downstream effects including the prevention of complications associated with postprandial hyperglycemia (especially in type 2 diabetics), including oxidative stress, chronic inflammation, etc., which were not measured in this study. 

While the phytochemical-rich ingredients used in our study per se did not give rise to any obvious effects on postprandial glycemia or insulinemia, there was a significant lowering of the postprandial insulinemic responses when the test meals were consumed within cooked rice (R) as compared to being consumed in the drink (D) form separately from the rice. This suggests that the food matrix in which phytochemical-rich ingredients are consumed has an effect on postprandial metabolic responses. Our research group has previously shown that both postprandial glycemic [22] and lipidemic [45] responses depended on the food matrix. Others have also shown that the postprandial glycemic lowering potential of polyphenol-rich ingredients depended on the form in which it was consumed [46], and various cooking methods were shown to significantly alter polyphenol bioaccessibility as well as the biological properties of polyphenol-rich ingredients, including differences in α-glucosidase inhibitory activity [47]. Furthermore, we and others have reported that the timing/sequence of consumption of polyphenol-rich ingredients (e.g., consumption prior to rather than concurrent consumption) produced different effects [48,49]. 

Similar to our findings, others have also reported greater postprandial insulinemic responses to a homogenized meal as compared with a solid meal [23]. There could be several mechanistic explanations for such observations. It is well known that various polyphenols can interact with starch molecules, leading to the formation of starch–polyphenol complexes [50,51] which can significantly restrict the access of digestive enzymes (e.g., amylases and glucosidases) and thereby the in vitro and in vivo digestibility of these starches [52,53,54]. Thus, the prior cooking of our ingredients within rice (R) may have led to differences in the extent of starch–polyphenol complex formations as well as starch accessibility and digestibility as compared to the ingredients being co-consumed within a separate drink (D). Moreover, we and others have previously shown that polyphenol-rich foods, including spices, can increase the secretion of gut hormones such as glucagon-like peptide-1 (GLP-1) [26,55]. It has also been shown previously that liquid meals (prepared by homogenizing solid meals) produced greater levels of both postprandial GLP-1 and insulin as compared to the same meal consumed solid [56]. Given that the gastric emptying of the liquid part of mixed (solid–liquid) meals occurs before the solid part [57], this may further explain the relatively greater insulinemic response when the ingredients were consumed within a drink (D) as compared to the same consumed within cooked rice (R). Given that postprandial insulinemia is an independent risk factor for diabetes and cardiovascular diseases [58,59], findings that food form plays a role has potential clinical implications. More research is therefore needed to confirm this observation. 

## 5. Conclusions

This study demonstrated that adding aqueous extracts of corn silk, cumin, and tamarind at dietary doses, in two separate forms, to a high-GI rice consisting of approximately 50 g of available carbohydrates in healthy volunteers conferred no additional benefits on postprandial glycemia or insulinemia. Our randomized, controlled clinical trial findings are insightful and advantageous in that these ingredients are traditionally believed in Asia to be beneficial in reducing the risk of diabetes, although to date, the majority of the previous evidence supporting these claims had been undertaken in animals, most often at relatively high doses. Some of the limitations of this study were the acute nature of the study design, the limited number of postprandial parameters being measured, and the fact that this study was undertaken in a cohort of healthy, normoglycemic, non-diabetic individuals. While other longer-term beneficial effects (e.g., anti-inflammatory, antioxidant, etc.) of the test ingredients in a population with a compromised glucose homeostasis (e.g., type 2 diabetics) cannot be ruled out, this study highlights the need for more controlled clinical trials in the future before specific claims are assumed regarding the specific benefits of traditional ingredients. Finally, this study also highlights the importance of the food form on postprandial insulinemia, in that phytochemical-rich ingredients in liquid forms (e.g., soups and beverages) may elicit greater postprandial insulinemic response than when consumed within a solid meal. Therefore, future applications of our study findings include the manipulation of food forms via various food consumption, preparation, and processing methods, in order to improve the metabolic consequences of certain foods and/or composited dishes.

## Figures and Tables

**Table 1:** Food ingredients, energy content, and nutrient composition of the test meals.

Treatment	Ingredients (per test meal)					Energy or Nutrients (per test meal)					
	Rice (g)	Peas (g)	Dried corn silk (g)	Cumin powder (g)	Tamarind pulp (g)	Energy (kcal)	Carbohydrate (g)	Protein (g)	Fat (g)	Fiber (g)	Available CHO (g)
**CON**	65	20	-	-	-	249.70	51.56	6.65	1.60	1.77	49.79
**CS12**	60	20	12	-	-	248.51	51.12	6.95	1.52	2.44	48.68
**CS24**	60	20	24	-	-	265.01	54.45	7.65	1.57	3.16	51.29
**CU**	60	20	-	24	-	288.16	53.53	8.29	4.26	4.11	49.42
**TAM**	60	20	-	-	20	259.00	52.79	6.87	1.98	2.13	50.65

CHO: carbohydrates; CON: control; CS12: corn silk 12 g; CS24: corn silk 24 g; CU: cumin; TAM: tamarind.

**Table 2:** Baseline anthropometric and metabolic characteristics.

Measurement	Mean ± S.D.
Age (Years)	37.5 ± 12.5
Height (m)	1.7 ± 0.06
Weight (kg)	63.9 ± 7.09
Body mass index (kg/m^2^)	21.8 ± 1.67
Body fat (%)	16.5 ± 4.25
Waist (cm)	78.8 ± 5.36
Systolic blood pressure (mmHg)	120.0 ± 7.75
Diastolic blood pressure (mmHg)	76.5 ± 7.37
Resting heart rate (bpm)	64. ± 10.03
Fasting blood glucose (mmol/L)	5.2 ± 0.43

**Table 3:** Incremental areas under the curve (iAUCs) for postprandial glucose and insulin over relevant time periods during various treatments.

Test Meal	iAUC 0–30 min		iAUC 0–120 min		iAUC 0–180 min	
	Glucose (mmol min/L)	Insulin (µU min/mL)	Glucose (mmol min/L)	Insulin (µU min/mL)	Glucose (mmol min/L)	Insulin (µU min/mL)
**Control** (plain rice with plain water)	36.55 ± 5.08	908.43 ± 141.98	158.06 ± 29.17	5598.56 ± 708.59	159.91 ± 29.59	6008.20 ± 751.33
**Test ingredients cooked in rice and consumed with plain water (R)**						
Corn silk (12 g)	36.15 ± 6.77	836.69 ± 128.20	147.99 ± 28.12	4802.84 ± 583.04	148.16 ± 28.11	5028.97 ± 633.59
Corn silk (24 g)	37.71 ± 4.18	839.87 ± 113.04	165.37 ± 30.87	4570.14 ± 543.70	171.41 ± 33.18	4890.21 ± 598.19
Cumin	44.12 ± 7.04	915.75 ± 106.42	193.05 ± 30.72	5223.44 ± 720.45	205.80 ± 33.69	5627.36 ± 817.57
Tamarind	38.97 ± 5.54	764.41 ± 110.33	181.02 ± 28.10	4503.82 ± 558.78	183.04 ± 28.37	4773.23 ± 591.69
**Test ingredients added into water and consumed with plain rice (D)**						
Corn silk (12 g)	40.98 ± 6.37	768.77 ± 101.19	220.90 ± 41.98	5138.30 ± 681.74	230.45 ± 45.09	5591.53 ± 723.78
Corn silk (24 g)	39.30 ± 5.37	944 ± 148.22	183.03 ± 29.20	5386.49 ± 698.89	186.99 ± 30.32	5729.12 ± 724.89
Cumin	38.54 ± 5.32	893.25 ± 96.40	167.52 ± 29.34	5768.09 ± 676.85	169.21 ± 29.96	6085.28 ± 714.54
Tamarind	39.32 ± 5.39	829.59 ± 98.12	182.05 ± 28.01	5679.60 ± 773.40	186.40 ± 29.89	6047.60 ± 814.98
***p*-Value for treatment (overall) ^a,b^**	0.912	0.499	0.404	0.080	0.310	0.051
***p*-Value for interaction ^a,c^**	0.632	0.601	0.143	0.669	0.091	0.721
***p*-Value for main effect of form ^a,c^**	0.973	0.647	0.274	0.005	0.329	0.003
***p*-Value for main effect of treatment ^a,c^**	0.932	0.176	0.983	0.247	0.985	0.227

^a^ All analyses were done using the linear mixed effects procedure with compound symmetry covariance structure in SPSS. ^b^ Model testing effects of treatment overall, including control. ^c^ Model testing effects of form × treatment without control. Bold lettering indicates *p* < 0.05.

**Table 4:** Baseline and total areas under the curve (tAUCs) of blood pressure over the postprandial period.

Treatment	Baseline (0 min)		Total AUC 0–180 min	
	Systolic BP (mmHg)	Diastolic BP (mmHg)	Systolic BP (mmHg min)	Diastolic BP (mmHg min)
**Control**	114.58 ± 3.12	71.56 ± 2.52	20,815.00 ± 528.18	12,902.50 ± 391.60
**Test ingredients cooked in rice and consumed with plain water (R)**				
**Corn silk (12 g)**	115.19 ± 2.25	72.81 ± 2.59	20,493.75 ± 458.67	11,454.17 ± 1049.87
**Corn silk (24 g)**	112.91 ± 3.16	72.91 ± 2.32	20,270.63 ± 526.11	11,385.83 ± 1034.56
**Cumin**	113.61 ± 2.37	73.22 ± 2.65	20,689.17 ± 471.23	12,929.17 ± 419.73
**Tamarind**	110.59 ± 3.11	70.0 ± 2.68	20,331.18 ± 487.74	12,169.17 ± 842.99
**Test ingredients added into water and consumed with plain rice (D)**				
**Corn silk (12 g)**	112.22 ± 2.57	72.0 ± 2.48	20,349.38 ± 448.14	11,344.17 ± 1030.44
**Corn silk (24 g)**	115.25 ± 2.46	73.41 ± 2.07	20,701.88 ± 511.97	11,649.17 ± 1046.57
**Cumin**	113.69 ± 2.74	72.61 ± 2.17	20,769.17 ± 448.02	13,054.17 ± 381.35
**Tamarind**	115.88 ± 2.44	72.06 ± 1.84	20,777.65 ± 365.70	12,315.83 ± 801.08
***p*-Value for treatment (overall)**	0.206	0.705	0.624	0.393
